# Probing Dynamic Self‐Reconstruction on Perovskite Fluorides toward Ultrafast Oxygen Evolution

**DOI:** 10.1002/advs.202201916

**Published:** 2022-07-22

**Authors:** Jing Zhang, Yu Ye, Zhenbin Wang, Yin Xu, Liangqi Gui, Beibei He, Ling Zhao

**Affiliations:** ^1^ Faculty of Materials Science and Chemistry China University of Geosciences Wuhan 430074 China; ^2^ State Key Laboratory of Geological Processes and Mineral Resources China University of Geosciences Wuhan 430074 China; ^3^ Department of Physics Technical University of Denmark Kongens Lyngby 2800 Denmark; ^4^ School of Physical and Mathematical Sciences Nanyang Technological University 21 Nanyang Link Singapore 637371 Singapore; ^5^ Shenzhen Research Institute China University of Geosciences Shenzhen 518000 China

**Keywords:** operando Raman tracking, oxygen evolution reaction, perovskites, self‐reconstruction, X‐ray absorption spectroscopy

## Abstract

Exploring low cost, highly active, and durable electrocatalysts for oxygen evolution reaction (OER) is of prime importance to boost energy conversion efficiency. Perovskite fluorides are emerging as alternative electrocatalysts for OER, however, their intrinsically active sites during real operation are still elusive. Herein, the self‐reconstruction on newly designed Ni—Fe coupled perovskite fluorides during OER process is demonstrated. In situ Raman spectroscopy, ex situ X‐ray absorption spectroscopy, and theoretical calculation reveal that Fe incorporation can significantly activate the self‐reconstruction of perovskite fluorides and efficiently lower the energy barrier of OER. Benefiting from self‐reconstruction and low energy barrier, the KNi_0.8_Fe_0.2_F_3_@nickel foam (KNFF2@NF) electrocatalyst delivers an ultralow overpotential of 258 mV to afford 100 mA cm^−2^ and an excellent durability for 100 h, favorably rivaling most the state‐of‐the‐art OER electrocatalysts. This protocol provides the fundamental understanding on OER mechanism associated with surface reconstruction for perovskite fluorides.

## Graphical abstract

Probing dynamic self‐reconstruction and establishing the reconstruction–property relationship on perovskite fluorides are investigated for oxygen evolution reaction (OER) in alkaline media. Fe incorporation accelerates the surface reconstruction of perovskite fluorides and reduces the OER activation energy. The resulting KNi_0.8_Fe_0.2_F_3_@nickel foam electrocatalyst demonstrates an extraordinary OER performance with ultralow overpotential and robust durability.

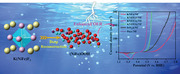

## 1 Introduction

Growing threats of energy shortage and global warming have stimulated intensive interest on renewable energy technologies.^[^ ^1^ ^]^ Electrochemical oxygen evolution reaction (OER) involving the sluggish four proton‐coupled electron transfer process becomes a bottleneck issue for a series of emerging energy technologies, for example, water splitting and rechargeable metal–air batteries.^[^ ^2^ ^]^ Therefore, accelerating the kinetics and gaining the fundamental understanding of OER are urgently required to realize energy‐efficient water oxidation. Up to now, iridium and ruthenium oxides with high intrinsic activities are recognized as the benchmark electrocatalysts for OER.^[^ ^3^ ^]^ Unfortunately, terrestrial scarcity, expensive cost, and inferior durability of those precious metal‐based electrocatalysts seriously restrict their broad commercialization in sustainable energy technologies.

To enable efficient and economical water oxidation, one of the most well‐established approaches involves the pursuit of alternative transition metal‐based electrocatalysts toward OER, including oxides,^[^ ^4^ ^]^ (oxy)hydroxides,^[^ ^5^ ^]^ phosphides,^[^ ^6^ ^]^ sulfides,^[^ ^7^ ^]^ et al. Currently, significant progresses have been achieved in the research and development of electrocatalysts. Amidst these candidates, perovskite materials (general formula of ABX_3_) are endowed with the unique properties of intrinsic 3D electronic/ionic diffusion channels, compositional flexibility, electronic structure tunability, and simple preparation. For instance, Ba_0.5_Sr_0.5_Co_0.8_Fe_0.2_O_3−_ *_*δ*_* (BSCFO)^[^ ^8^ ^]^ perovskite oxide and PrBaCo_2_O_5+_ *_*δ*_* (PBCO)^[^ ^9^ ^]^ layered perovskite oxide demonstrated higher intrinsic OER activities than that of the benchmark IrO_2_ electrocatalyst. Subsequently, remarkable strategies, including morphology designing,^[^ ^10^ ^]^ phase engineering,^[^ ^11^ ^]^ defect tailoring,^[^ ^12^ ^]^ surface reconstruction,^[^ ^13^ ^]^ heterostructure constructing,^[^ ^14^ ^]^ et al., have been implemented to substantially boost the electrocatalytic activity of perovskite oxides. For the underlying mechanism of OER, several inherent indicators regarding the activity origin of perovskite oxides have been revealed, such as e_g_‐occupancy,^[^ ^8^ ^]^ metal‐oxygen covalency,^[^ ^15^ ^]^ lattice oxygen participation,^[^ ^16^ ^]^ and oxygen vacancy,^[^ ^12^, ^17^ ^]^ etc. Among them, the partial substitution of F anion into perovskite oxides could trigger the surface oxygen for catalyzing OER.^[^ ^12d^ ^]^

Besides perovskite oxides and perovskite oxyfluorides, perovskite fluorides are developed as a new platform of potential electrocatalysts toward OER. Dai's group, for the first time, demonstrated that high‐entropy perovskite fluorides were electrochemically active to drive OER.^[^ ^18^ ^]^ The K_0.8_Na_0.2_(MgMnFeCoNi)F_3_ electrocatalyst delivered a low overpotential of 314 mV to achieve 10 mA cm^−2^ for OER, better than the benchmark IrO_2_ electrocatalyst. In addition, the structure of such high‐entropy perovskite fluoride almost retained after a long‐term OER operation. Interestingly, in view of the strongest electronegativity of F anion, a weaker metal‐F bond tends to be formed in fluorides relative to the metal‐O bond in oxides. The metal‐F bond with weaker dissociation readily induces stronger ionicity for driving surface reconstruction.^[^ ^19^ ^]^ For example, dynamic reconstruction with a formation of F incorporated CoOOH was identified on the CoF_2_ surface, resulting in a remarkably enhanced OER activity.^[^ ^19a^ ^]^ Similar surface reconstruction behavior were also confirmed on Fe—Co—F nanocubes,^[^ ^19d^ ^]^ quasi‐single‐crystalline cobalt fluoride nanorods,^[^ ^19e^ ^]^ (FeNiCo)F_2_ layer,^[^ ^19f^ ^]^ Fe‐doped Ni oxyfluoride layer,^[^ ^19g^ ^]^ and F incorporated NiFe hydroxide.^[^ ^19h^ ^]^ It has been disclosed that surface reconstruction on metal fluorides has great impacts on real active sites and intrinsic catalytic activities toward OER. Due to the different coordination structure, engineering and stimulating self‐reconstruction behavior on perovskite fluorides is another story, such as KNi_1−_ *_x_* Co*_x_* F_3_.^[^ ^20^ ^]^ Nevertheless, to the best of our knowledge, probing dynamic self‐reconstruction and establishing the reconstruction–property relationship on perovskite fluorides are rarely reported.

Motivated by this view, herein, we highlight KNi_1−_ *_x_* Fe*_x_* F_3_ (*X* = 0, 0.1, 0.2, 0.3, denoted as KNF, KNFF1, KNFF2, KNFF3) perovskite fluorides with dynamic surface reconstruction for efficiently catalyzing OER in alkaline media. In general, Fe incorporation can induce the significant enhancement in the OER activity of Ni based electrocatalysts owing to the synergetic effect between Ni and Fe.^[^ ^21^ ^]^ Therefore, Ni—Fe coupled perovskite fluorides are proposed as potential candidates to drive OER. These perovskite fluorides nanocubes are synthetized via a facile one‐pot hydrothermal method. Unveiled by operando Raman spectroscopy and ex situ X‐ray absorption spectroscopy (XAS), these newly designed perovskite fluorides undergo the self‐reconstruction facilitated via Fe incorporation during OER operation, generating electrochemically active metal (oxy)hydroxides. Impressively, the resultant KNFF2@nickel foam (KNFF2@NF) electrocatalyst demonstrates an extremely low overpotential of 258 mV to achieve a high current density of 100 mA cm^−2^, superior to benchmark IrO_2_ and most reported state‐of‐the‐art OER electrocatalysts. As a proof‐of‐concept, an assembled electrolyzer using the KNFF2‖Pt/C coupled electrocatalysts exhibits high electrochemical performance and robust durability. Theoretical calculations reveal that Fe incorporation not only facilitates the surface reconstruction of perovskite fluorides but also reduces the activation energy of OER kinetics. Engineering self‐reconstruction showcases a promising approach to rationally design advanced electrocatalysts for sustainable energy technologies.

## 2 Results and Discussion

### 2.1 Electrocatalysts Characterization

**Figure 1:**
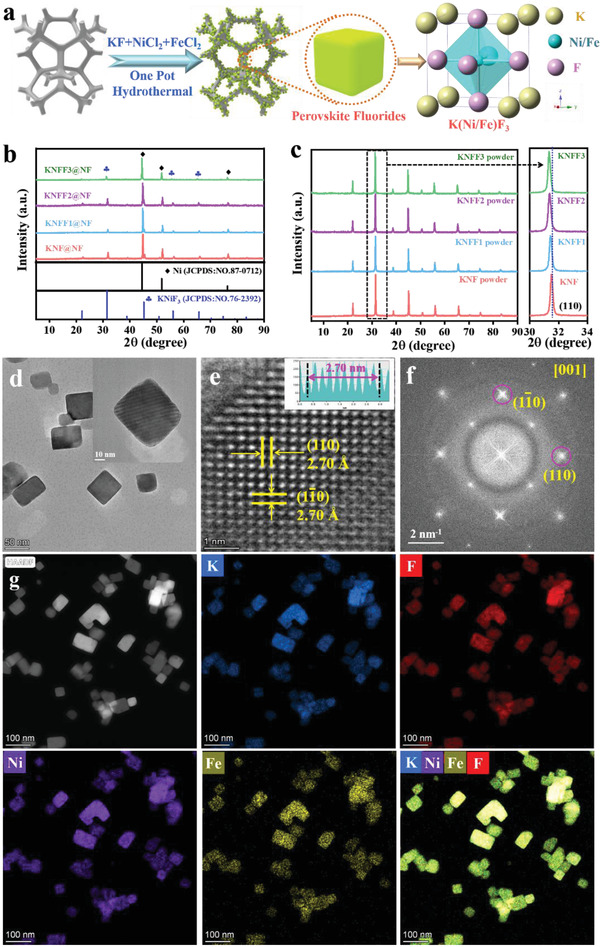
a) Illustration of perovskite fluorides synthesis; b) XRD patterns of KNF@NF, KNFF1@NF, KNFF2@NF, KNFF3@NF electrocatalysts; c) XRD patterns of KNF, KNFF1, KNFF2, KNFF3 powders; d) low magnification, e) high magnification HRTEM images; f) Fast flourier transform; and g) EDS elemental mapping of KNFF2 electrocatalyst.

As illustrated in **Figure** **1**a, the synthesis of Ni—Fe coupled perovskite fluorides on nickel foam (NF) were achieved via a facile one step hydrothermal process. Figure 1b shows the X‐ray diffraction (XRD) patterns of KNF@NF, KNFF1@NF, KNFF2@NF, and KNFF3@NF electrocatalysts. It is pellucid that all as‐synthesized electrocatalysts adopt a cubic perovskite structure (KNF, JCPDS: NO. 76–2392) and a metallic Ni phase of NF matrix, suggesting the successful preparation of perovskite fluorides on NF. To eliminate the influence of NF, the fluoride powders were further stripped from NF for XRD analysis. As depicted in Figure 1c, with the increase of Fe concentration from 0 to 0.3, the representative diffraction peak of (110) plane for perovskite fluorides gradually shifts to the reduced angles, denoting that the lattice expansion of perovskite fluorides is induced by Fe incorporation. In addition, Table S1, Supporting Information, illustrates the measured molar ratios of Ni:Fe in the as‐prepared perovskite fluorides, determined by inductively coupled plasma optical emission spectrometer (ICP‐OES).

Scanning electron microscopy (SEM) images in Figure S1, Supporting Information, exhibit that the surface of pristine NF is clean and smooth. After one‐pot hydrothermal process, those perovskite fluorides deposited on NF surface adopt the morphology of 3D nanocubes with the average particle size of ≈50 nm (Figure S2, Supporting Information). After the substitution of Fe, the morphologies of perovskite fluorides have no significant changes. High resolution transmission electron microscopy (HRTEM) was further carried out to receive an insight into the microstructure of KNFF2 perovskite. Figure 1d reconfirms the nanocubes architecture of KNFF2 perovskite. As shown in Figure 1e, the obtained lattice spacings of KNFF2 perovskite are 2.70 Å, well indexed to its (110) and (1‐10) crystal planes derived from XRD analysis. Additionally, the fast Fourier transform pattern fairly agrees with the [001] zone axes of KNFF2 cubic perovskite (Figure 1f). In addition, elemental mapping shows that the K, Ni, Fe, and F elements are uniformly distributed in the as‐prepared KNFF2 perovskite (Figure 1g).

### 2.2 Electrocatalytic OER Performance

**Figure 2:**
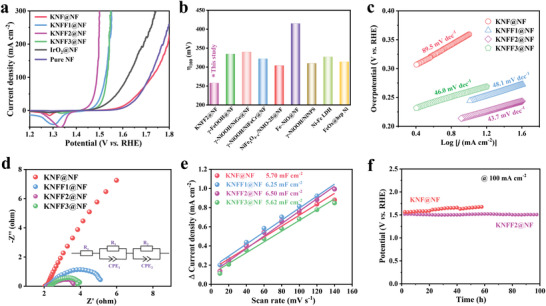
OER performance of KNF@NF, KNFF1@NF, KNFF2@NF, and KNFF3@NF electrocatalysts in 1.0 m KOH. a) LSV polarization curves; b) comparison of overpotentials at 100 mA cm^−2^; c) Tafel plots; d) Nyquist plots; e) double‐layer capacitances; and f) long‐term chronopotentiometry curves.

To assess the OER performance of perovskite fluorides, the KNF@NF, KNFF1@NF, KNFF2@NF, KNFF3@NF and commercial IrO_2_@NF electrocatalysts were measured in O_2_‐saturated 1.0 m KOH electrolyte based on a standard three‐electrode cell. As displayed in linear sweep voltammetry (LSV) curves (**Figure** **2**a), prior to the typical OER peaks, all perovskite fluorides possess distinct redox peaks, probably originating from the electrochemical transformation between Ni^II^ and Ni^III^. As revealed, Fe incorporation suppresses the electrochemical transformation between Ni species, resulting in the shift of the redox peaks in LSV.^[^ ^22^ ^]^ Moreover, the Fe incorporated perovskite fluorides@NF electrocatalysts exhibit substantially improved OER activities relative to the pristine KNF@NF electrocatalyst. The optimized KNFF2@NF electrocatalyst offers a lower overpotential of only 258 mV to achieve 100 mA cm^−2^ than 494 mV for KNF@NF, 295 mV for KNFF1@NF, for 301 mV KNFF3@NF, 390 mV for IrO_2_@NF and 485 mV for pristine NF, indicative of the highest electrocatalytic OER activity among these studied electrocatalysts. Of note, the ultralow overpotential on KNFF2@NF electrocatalyst favorably outperforms most recently reported OER electrocatalysts (e.g., *γ*‐FeOOH@NF,^[^ ^23^ ^]^ NiOOH/NiGe@NF,^[^ ^24^ ^]^ NiOOH/NiFeCr@NF,^[^ ^25^ ^]^ NiFe_2_O_4−_ *_x_*/NMO‐25@NF,^[^ ^26^ ^]^ Fe—NiO@NF,^[^ ^27^ ^]^ *γ*‐NiOOH/NiNPS,^[^ ^28^ ^]^ Ni—Fe LDH,^[^ ^29^ ^]^ FeO*_x_* @hcp Ni,^[^ ^30^ ^]^ Figure 2b and Table S2, Supporting Information), demonstrating an outstanding OER electrocatalysis achieved on KNFF2@NF electrocatalyst.

Furthermore, the KNFF2@NF electrocatalyst delivers the smallest Tafel slope of 43.7 mV dec^−1^ among those perovskite fluorides (Figure 2c), suggestive of the most rapid OER kinetics. In the case of electrochemical impedance spectra (EIS) evaluations (Figure 2d), the equivalent circuit of *R*_o_(*R*_1_CPE_1_)(*R*_2_CPE_2_) is applied for EIS fitting, where *R*_o_, *R*_1_ and *R*_2_ represent the solution resistance, electrode texture resistance and charge transfer resistance, respectively.^[^ ^31^ ^]^ The KNFF2@NF electrocatalyst presents a smaller charge transfer resistance of 1.39 Ω than those of other perovskite fluorides (Table S3, Supporting Information), implying a faster charge transfer kinetics. To evaluate the effective electrochemical surface area (ECSA), cyclic voltammetry (CV) curves at different scan speeds ranging from 10 to 140 mV s^−1^ were conducted, as shown in Figure S3, Supporting Information. Derived from CV curves, the fitted double layer capacitances (*C*_dl_) of perovskite fluorides@NF are illustrated in Figure 2e. The KNFF2@NF electrocatalyst provides a higher *C*_dl_ (6.50 mF cm^−2^) than those of KNF@NF (5.70 mF cm^−2^), KNFF1@NF (6.25 mF cm^−2^) and KNFF3@NF (5.62 mF cm^−2^), denoting that more electrochemically active sites on the KNFF2@NF electrocatalyst. Figure S4, Supporting Information, provides the LSV curves normalized by electrochemical surface area (ECSA). The KNFF2@NF electrocatalyst still exhibits the highest specific current density among those perovskite fluorides, suggestive of the best intrinsic OER activity. As known, durability is a key indicator for the practical application of electrocatalysts. To monitor the stability of electrocatalyst, the chronopotentiometry measurements were conducted on KNF@NF and KNFF2@NF electrocatalysts under OER conditions. Compared to KNF@NF, Fe incorporated KNFF2@NF delivers an enhanced stability for OER (Figure 2f). The potential required to achieve a current density of 100 mA cm^−2^ over the KNFF2@NF electrocatalyst is ≈1.51 V (versus reversible hydrogen electrode [RHE]) and almost retains around such potential for 100 h, demonstrating a highly efficient and stabilized water oxidation electrocatalysis.

**Figure 3:**
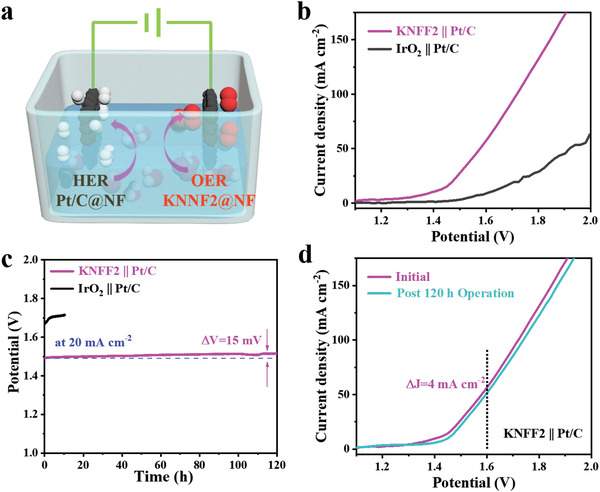
Overall water splitting application in 1.0 m KOH. a) Schematic diagram; b) polarization plots and c) chronopotentiometry curves of KNFF2||Pt‐C and IrO_2_||Pt/C based electrolyzers; and d) polarization plots of KNFF2||Pt‐C based electrolyzer before and after long‐term operation.

Encouraged by high activity and good stability of KNFF2@NF electrocatalyst, the homemade electrolysis cell consisting of KNFF2@NF anode, 1.0 m KOH electrolyte and Pt/C@NF cathode was assembled for overall alkaline water splitting (**Figure** **3**a). Besides, the IrO_2_||Pt/C based electrolyzer was also assembled for comparison. Notably, the polarization curves in Figure 3b exhibit that the KNFF2||Pt‐C based electrolyzer requires only 1.40 V to achieve the current density of 10 mA cm^−2^, superior to the benchmark IrO_2_||Pt/C based electrolyzer (1.61 V to reach 10 mA cm^−2^). In addition, the electrolyzer with KNFF2||Pt‐C electrocatalysts almost remains stable during real water splitting for 120 h, displaying a voltage decline of only 15 mV at 20 mA cm^−2^ (Figure 3c) and a current density degeneration of 4 mA cm^−2^ at 1.60 V (Figure 3d). This electrolysis cell evaluation confirms the superiority and feasibility of KNFF2 electrocatalyst for practical water oxidation. For the stability of IrO_2_||Pt/C based electrolyzer, the cell performance declines seriously during the initial 10 h operation owing to the dissolution of commercial IrO_2_ even in alkaline media.

### 2.3 Dynamic Surface Reconstruction

**Figure 4:**
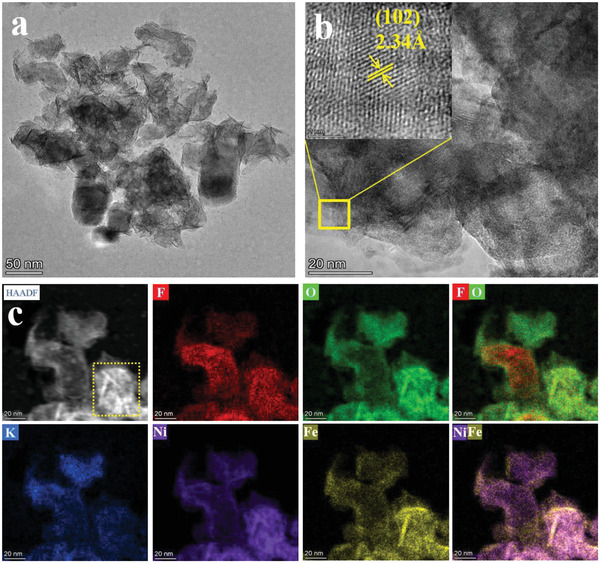
Microstructure of KNFF2 electrocatalyst after OER operation. a) Low magnification and b) high magnification HRTEM images; and c) EDS elemental mapping.

It is well documented that the metal fluorides with weak interactions and easy dissociation readily suffer from surface reconstruction.^[^ ^19i^ ^]^ In view of the different crystal structure, dynamic self‐reconstruction on metal perovskite fluorides is not yet understood. To unveil the real electrochemically active sites of perovskite fluorides for OER, the morphology and the phase evolution of KNFF2@NF electrocatalyst after OER operation were intensively investigated. As revealed via XRD, the phase of KNFF2 perovskite is predominantly transformed to Fe incorporated (NiFe)(OH)_2_ and *γ*‐(NiFe)OOH after OER process (Figure S5, Supporting Information). Whereas, the perovskite structure of KNF almost retains with less formation of (oxy)hydroxide after OER test. Therefore, we can speculate that the Fe incorporation in perovskite fluorides promotes a self‐reconstitution behavior to create rich (oxy)hydroxide species. Additionally, the relatively small peak of *γ*‐(NiFe)OOH for KNFF2 post‐OER might be attributed to the poor crystallinity of electrochemically‐created (NiFe)OOH. Examined by SEM, the reconstructed KNFF2 (Figure S6, Supporting Information) and KNF (Figure S7, Supporting Information) surfaces become coarse and porous in nanoscale after OER operation. We further conducted HRTEM on the KNFF2 post‐OER electrocatalyst to shed light on the reconstructed morphology. As described in **Figure** **4**a, the KNFF2 nanocubes are rapidly reconstructed, generating a number of nanosheets covered on the perovskite matrix. Importantly, such highly porous nanosheets ensure a high specific surface area that can expose rich active sites. The observed interplanar distance of 2.34 Å is fairly assigned to the (102) plane of *γ*‐(NiFe)OOH (Figure 4b). In addition, elemental mapping images of KNFF2 post‐OER exhibit that the elements of Ni, Fe, O rather than K, F are enriched in the marked reconstructed nanosheets (Figure 4c). On the other hand, a moderate reconstruction layer of (oxy)hydroxide is confirmed on the KNF surface after OER operation (Figure S8, Supporting Information), obviously distinguishing itself from the deep reconstruction on KNFF2 perovskite.

Surface analysis by X‐ray photoelectron spectroscopy (XPS) further confirms the phase evolution after OER operation. As shown in Figure S9a, Supporting Information, the Ni^2+^ 2p_3/2_ peak located at 857.9 eV for the as‐prepared KNFF2 perovskite is well indexed to the reported Ni—F bond.^[^ ^32^ ^]^ As a contrast, the Ni^2+^ 2p_3/2_ peak for the KNFF2 post‐OER shifts to a lower binding energy of 855.7 eV, corresponding to the created (NiFe)(OH)_2_. Besides, a Ni^3+^ 2p_3/2_ peak at 857.3 eV is also obtained after OER test probably due to the existence of *γ*‐(NiFe)OOH.^[^ ^33^ ^]^ Likewise, the Ni^2+^ 2p_3/2_ and Ni^3+^ 2p_3/2_ peaks are observed over KNF electrocatalyst after OER operation (Figure S10a, Supporting Information), implying the possible co‐existence of Ni(OH)_2_ and *γ*‐NiOOH. Regarding the Fe 2p spectra for KNFF2, the Fe^2+^ 2p_3/2_ peak located at 712.0 eV for the pristine KNFF2 perovskite corresponds to Ni—F bond.^[^ ^34^ ^]^ After the reconstruction process, the Fe^2+^ 2p_3/2_ and Fe^3+^ 2p_3/2_ peaks are obtained, agreeing with the formation of (oxy)hydroxide species as well.^[^ ^35^ ^]^ In the case of O 1s spectrum for the post‐OER KNFF2 (Figure S9c, Supporting Information), the obtained metal‐O peak located at 530.5 eV and metal‐OH peak located at 531.3 eV imply the formation of (oxy)hydroxide species as well.

**Figure 5:**
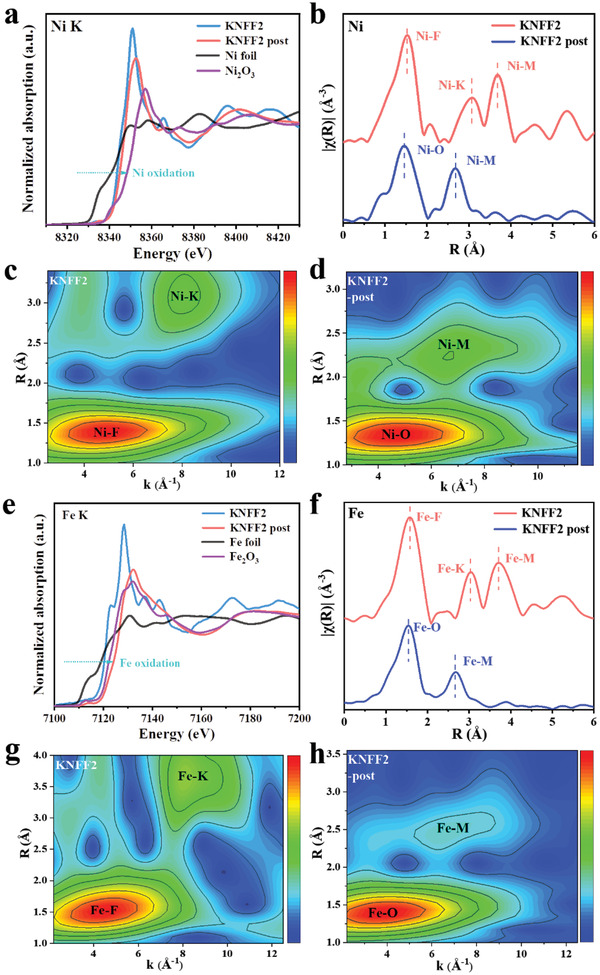
XANES spectra of a) K‐edge; b) Fourier transform EXAFS spectra; wavelet transform for Ni in c) KNFF2 and d) KNFF2 post‐OER; XANES spectra of e) K‐edge; f) Fourier transform EXAFS spectra; and wavelet transform for Fe in g) KNFF2 and h) KNFF2 post‐OER.

We conducted X‐ray absorption near‐edge structure (XANES) spectroscopy and extended X‐ray absorption fine structure (EXAFS) spectroscopy, to investigate the electronic structure and coordination structure of electrocatalysts before and after an OER operation. As depicted in **Figure** **5**a, the Ni K‐edge of XANES spectra on KNFF2 has a positive shift of 1.5 eV after OER operation, indicating the partial oxidation state of Ni^3+^ in the KNFF2 post‐OER electrocatalyst. This valence state change of Ni fairly agrees with the previous XPS results. Figure 5b presents the Fourier transformation EXAFS spectra of Ni. For the KNFF2 perovskite, the first shell peak at 2.00 Å represents Ni—F bond. The coordination number of Ni—F is ≈6.0 (Table S4, Supporting Information), demonstrating typical octahedral coordination in perovskite structure. Moreover, two other obvious peaks at 3.09 and 3.68 Å (not processed) are assigned to Ni—K and Ni—M (M = Ni or Fe) paths. In the case of the KNFF2 post‐OER, the Ni—O path becomes the first shell with the fitted bond distance of 2.00 Å, and the Ni—M path is the second shell with the fitted bond distance of 2.67 Å (not processed). This changed Ni K‐edge is likely ascribed to the creation of self‐reconstructed oxyhydroxide. Similar to Ni K‐edge, the existence of Fe^3+^ in KNFF2 post‐OER is confirmed in Figure 5e, where the Fe K‐edge is close to the standard Fe_2_O_3_. The typical shells of Fe are almost consistent with Ni shells (Figure 5f), indicative of Fe incorporation in both the pristine KNFF2 before OER and the reconstructed oxyhydroxide after OER. Figure 5c,d,g,h presents the wavelet transform for Ni in KNFF2 and KNFF2 post‐OER and Fe in KNFF2 and KNFF2 post‐OER, respectively. In particular, the maxima of Ni—O and Ni—M (Figure 5d) have similar wave numbers and distances relative to the maxima of Fe—O and Fe—M (Figure 5h) in the wavelet transform of the KNFF2 post‐OER, implying that both Ni and Fe are located in the similar coordination environment. The detailed fitting curves of the Ni and Fe corresponding structure for KNFF2 post‐OER in R space and k space are, respectively, shown in Figures S11 and S12, Supporting Information. These XANES and EXAFS results evidentially illustrate that the KNFF2 perovskite is self‐reconstructed as a formation of (NiFe)OOH during OER process.

**Figure 6:**
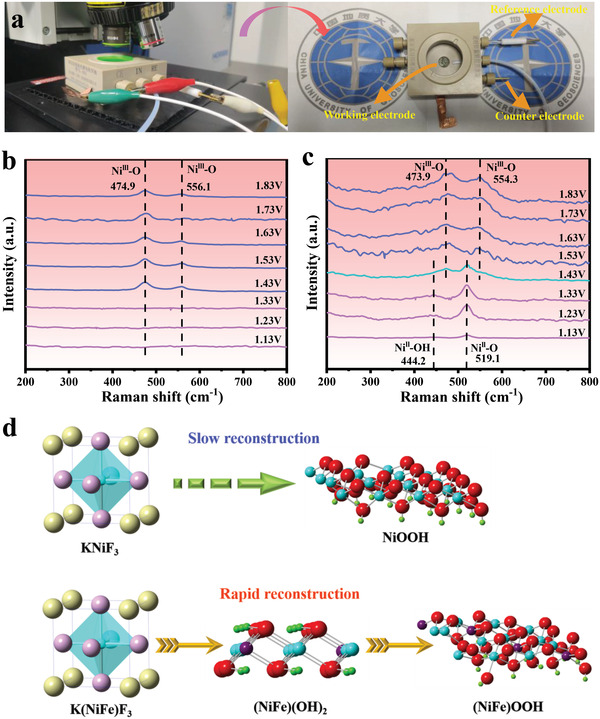
a) Schematic diagram of in situ Raman device; in situ Raman spectra on b) KNF@NF and c) KNFF2@NF electrocatalysts at applied potentials from 1.13 to 1.83 V; and d) proposed model of surface reconstruction on perovskite fluorides during OER process.

Ex situ Raman spectra of KNFF1@NF, KNFF2@NF, and KNFF3@NF were primarily performed before and after OER operation. Compared to the as‐prepared electrocatalysts, the KNFF1@NF, KNFF2@NF, and KNFF3@NF electrocatalysts post‐OER exhibit the unique peaks of 473.9 and 554.3 cm^−1^ (Figure S13, Supporting Information), providing solid evidence of the existence of *γ*‐(NiFe)OOH after OER process.^[^ ^36^ ^]^ To reveal the underlying dynamic surface reconstruction that occurred during the OER electrocatalysis, the in situ Raman spectra were subsequently carried out with the potential ranging from 1.13 to 1.83 V (versus RHE). The home‐assembled in situ Raman device combining Raman detector and open spectro‐electrochemical cell is depicted in **Figure** **6**a. In the case of KNF@NF electrocatalyst (Figure 6b), no obvious Raman peaks are obtained on KNF@NF when the potential is below 1.43 V. Further improving the potential, the Raman peaks assigned to the stretching vibrations of *γ*‐NiOOH are emerging, indicating that the surface reconstruction occurs until the potential achieves at 1.43 V. By contrast, Fe incorporated KNFF2@NF electrocatalyst undergoes surface reconstruction even at the low potential of 1.13 V with the formation of Ni(OH)_2_ (Figure 6c). Furthermore, the electrochemical transformation from (NiFe)(OH)_2_ to *γ*‐(NiFe)OOH is confirmed when the potential reaches at 1.43 V. According to the above in situ Raman findings, the reconstruction evolution of KNFF2 electrocatalyst is likely NiFe‐coupled perovskite fluoride‐(NiFe)(OH)_2_‐*γ*‐(NiFe)OOH along with the increased potential toward OER, the corresponding schematic diagram of which is illustrated in Figure 6d. Notably, the signals of *γ*‐(NiFe)OOH on KNFF2@NF electrocatalyst are significantly higher than those on KNF@NF electrocatalyst, revealing a deeper reconstruction occurred on KNFF2@NF electrocatalyst. Thereby, we can speculate that Fe incorporation substantially enables the rapid self‐reconstruction of Ni—Fe coupled perovskite fluorides, resulting in the abundant electrochemically active sites of (oxy)hydroxides to efficiently catalyze OER. In addition, the in situ Raman spectra of KNFF2 electrocatalyst during long‐term OER operation at 1.53 V versus a RHE are depicted in Figure S14, Supporting Information. The ever present (oxy)hydroxides contribute to the high durability of KNFF2 electrocatalyst for OER.

### 2.4 Reconstruction and OER Mechanism

**Figure 7:**
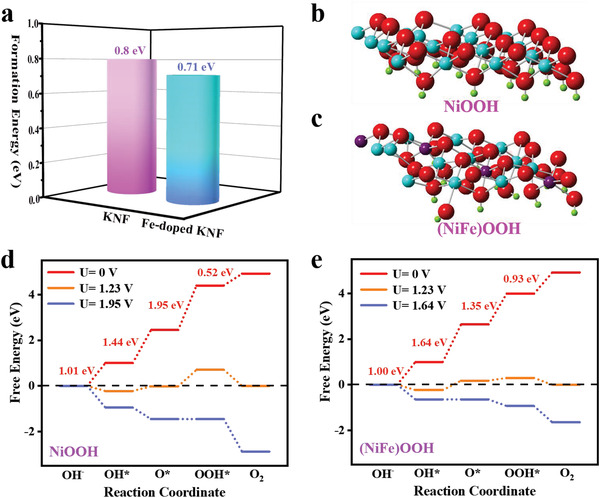
DFT calculations. a) Formation energies of F vacancy for on KNF and Fe‐doped KNF electrocatalysts; schematic of reconstructed b) NiOOH surface and c) (NiFe)OOH surface (Ni atom: cyan, Fe atom: purple, O atom: red, H atom: green); and free‐energy diagrams for alkaline OER pathways on d) NiOOH surface and e) (NiFe)OOH surface.

With the aid of density functional theory (DFT) calculation, the influence of Fe incorporation on surface reconstruction and OER mechanism of perovskite fluorides were intensively studied. As mentioned above, the reconstruction process with (oxy)hydroxides formation is a prerequisite to efficiently drive OER. As revealed, the self‐reconstruction on F^−^‐incorporated NiFe hydroxide was induced via F leaching under OER operation.^[^ ^19h^ ^]^ In perovskite series, the vacancy formation energy of anion is also regarded as a crucial indicator for the inner driving force of reconstruction.^[^ ^37^ ^]^ Considering that the (100) surface is the most stable surface of perovskite fluorides, the perfect (100) surface and F defective (100) surface for KNF (Figure S15, Supporting Information) and KNFF2 (Figure S16, Supporting Information) are built. **Figure** **7**a shows that the calculated formation energy of F vacancy (*E*_Fv_) for KNF and KNFF2 are respectively 0.80 and 0.71 eV, denoting that the formation of F vacancy on KNFF2 (100) surface is energetically more favorable than that on KNF (100) surface. Consequently, this facilitated formation of F vacancy triggered by Fe incorporation is instrumental in driving surface reconstruction.

Based on a widely accepted mechanism for the alkaline OER,^[^ ^38^ ^]^ the electrocatalysis abides by the following four proton/electron‐coupled steps:

(1) $$\mathrm{Step}1:\;\;{}^{*}+OH^{-}\to OH^{*}+e^{-}$$

(2) $$\mathrm{Step}2:\;\;{\mathrm{OH}}^{*}+{\mathrm{OH}}^{-}\to O^{*}+H_{2}O+e^{-}$$

(3) $$\mathrm{Step}3:\;\;O^{*}+OH^{-}\to \mathrm{OO}H^{*}+e^{-}$$

(4) $$\mathrm{Step}4:\;\;\mathrm{OO}H^{*}+OH^{-}\to O_{2}{+}^{*}+H_{2}O+e^{-}$$

where * represents an active site on the surface of electrocatalyst. OH*, O*, and OOH* are the reaction intermediates. The oxygen reaction intermediates readily adsorb on surface Ni (or Fe) sties, due to the preferential interaction of Ni (or Fe) 3d orbital with O 2p orbital (oxygen intermediates).^[^ ^39^ ^]^ Figure 7b,c provides the schematic diagrams of electrochemically active *γ*‐NiOOH and *γ*‐(NiFe)OOH, respectively, derived from the surface reconstruction on KNF and KNFF2. The OER process involving reaction intermediates on *γ*‐NiOOH and *γ*‐(NiFe)OOH surfaces are further described in Figures S17 and S18, Supporting Information, respectively. The calculated free energies of four elementary reactions on reconstructed *γ*‐NiOOH and *γ*‐(NiFe)OOH are presented in Figure 7d,e. We can see that the OOH* formation is the potential determining step over *γ*‐NiOOH surface with the calculated overpotential of 0.72 eV. In contrast, the O* formation is the potential determining step over *γ*‐(NiFe)OOH surface with the calculated overpotential of 0.41 eV. Therefore, it is concluded that Fe incorporation into *γ*‐NiOOH not only alters the OER mechanism but also mitigates the energy barrier for OER kinetics, ultimately contributing to intrinsic OER activity. Furthermore, several studies had also revealed that the activity trend of Ni—Fe coupled oxyhydroxide as a function of Fe incorporation showed a volcano plot. For Fe levels below ≈25%, Fe^3+^ incorporated into (NiFe)OOH lattice with short Fe−O bond delivered the near optimal adsorption energies of OER intermediates and the low overpotentials at Fe sites. As the Fe level rose above 25%, the inactive FeOOH phase became predominant.^[^ ^40^ ^]^

## 3 Conclusions

In summary, we reveal the dynamic self‐reconstruction on novel Ni—Fe coupled perovskite fluorides and correlated the structural evolution with intrinsic OER activity. As demonstrated by in situ Raman spectra, ex situ XAS spectra and DFT calculation, the self‐reconstruction of perovskite fluorides is triggered by Fe incorporation, and the resultant Fe incorporated *γ*‐(NiFe)OOH delivers a reduced activation energy for OER relative to the pristine *γ*‐NiOOH. Benefiting from the self‐reconstruction with the formation of electrochemically active *γ*‐(NiFe)OOH, the KNFF2@NF electrocatalyst exhibits an extraordinary OER performance with an ultralow overpotential of 258 mV to achieve 100 mA cm^−2^ and a long‐lasting stability for 100 h. As an application, the KNFF2||Pt‐C based electrolyzer requires only 1.40 V to achieve the current density of 10 mA cm^−2^ and almost remains stable for a 120 h operation, superior to the commercial IrO_2_||Pt/C based electrolyzer. These insights open possibilities for engineering self‐reconstructed electrocatalysts for multiple electrocatalysis.

## 4 Experimental Section

### Electrocatalyst Synthesis

Potassium fluoride dihydrate (KF·2H_2_O), nickel chloride hexahydrate (NiCl_2_·6H_2_O), iron chloride tetrahydrate (FeCl_2_·4H_2_O), ethylene glycol, ethanol, hydrochloric acid (HCl), and potassium hydroxide (KOH) were analytically pure and were purchased by Sinopharm Group. Nickel foam (NF), Nafion solution, commercial Pt/C catalyst (loading 20% Pt), and commercial IrO_2_ powder were provided by Sigma Aldrich. NF (6 cm × 2 cm) was purified by the sonication in 6 m HCl for 10 min and then in ethanol for 30 min before synthesis.

Perovskite fluorides, KNiF_3_ (denoted as KNF), KNi_0.9_Fe_0.1_F_3_ (denoted as KNFF1), KNi_0.8_Fe_0.2_F_3_ (denoted as KNFF2) and KNi_0.7_Fe_0.3_F_3_ (denoted as KNFF3), were grown on NF via a one step hydrothermal way. Taking the preparation of KNFF2@NF for example, 9.6 mmol NiCl_2_·6H_2_O, 2.4 mmol FeCl_2_·4H_2_O, 36 mmol KF·2H_2_O and 30 mL ethylene glycol were stirred in a beaker for 30 min. Subsequently, this homogeneous solution together with the bare NF were transferred into a 50 mL Teflon‐lined stainless autoclave, followed by heat treatment at 180 °C for 20 h to obtain the KNFF2@NF electrocatalyst. Other perovskite fluorides with corresponding stoichiometry were prepared on NF under the same conditions. For comparison, IrO_2_ ink, consisting of 80 mg of commercial IrO_2_ powder, 19.5 mL of ethanol and 0.5 mL Nafion solution, was dropped onto NF to gain IrO_2_@NF electrocatalyst. The mass loading of electrocatalysts on NF was ≈3.0 mg cm^−2^.

### Structural Characterization

X‐ray diffraction (XRD, Bruker, D8 Advance) with Cu K*α* radiation was carried out to analyze phase structures of electrocatalysts. Inductively coupled plasma optical emission spectrometer (ICP‐OES, Perkinelmer) was conducted to obtain chemical compositions of electrocatalysts. SEM and HRTEM images were taken on Hitachi SU‐8010 and Titan G260‐300, respectively, to observe the microstructures of the electrocatalysts. XPS (K‐ALPHA) using monochromatic Al K*α* excitation was conducted to identify surface electronic structures of electrocatalysts. For XAS, the catalysts and reference samples of Ni and Fe K‐edge were collected at the beamline BL17C1 at the National Synchrotron Radiation Research Center (NSRRC), Taiwan.

Raman spectroscopy (Horiba Jobin Yvon S.A.S.) was equipped with a frequency doubled Nd:YAG laser to achieve 532 nm excitation. Raman curves were recorded at 1800 lines/mm ranging from 10 to 2500 cm^−1^. Operando Raman spectra were performed using a homemade open spectro‐electrochemical cell consisting of a three‐electrode system. Self‐supported electrocatalyst, saturated calomel and Pt sheet served as the working electrode, the reference electrode and the counter electrode, respectively. All spectra were calibrated by referencing a standard silicon wafer to 520.5 cm^−1^.

### Electrochemical Characterization

Based on the standard three‐electrode setup, electrochemical tests were performed by a CHI 760E electrochemical workstation. The three‐electrode device was composed of the working electrode of synthesized electrocatalysts, the reference electrode of saturated calomel electrode (SCE), and the counter electrode of graphite rod. 1 m KOH aqueous solution was applied as the electrolyte. LSV and cyclic voltammetry (CV) curves were carried out at the scanning speed of 5 mV s^−1^. EIS were collected by applying an AC voltage of 10 mV amplitude with the frequencies ranging from 100 kHz to 0.1 Hz. Electrochemical double‐layer capacitors (*C*_dl_) were recorded via scanning CV at different scan rates in the potentials from 0.15 to 0.25 V versus a RHE. Chronopotentiometry were carried out to monitor the operation durability of electrocatalysts. All measured potentials were corrected with 85% iR correction, followed by the calibration versus the potential of a RHE.

### Theoretical Calculation

All the spin‐polarized DFT computations were performed via Vienna ab initio simulation package (VASP).^[^ ^41^ ^]^ To address the ion–electron interactions, the projector augmented wave method^[^ ^42^ ^]^ together with the general gradient approximation in the Perdew–Burke–Ernzerhof (PBE) form were adopted.^[^ ^43^ ^]^ Given the fact that the (100) crystal plane was the most stable surface for the cubic perovskite fluorides, we thus built a (100) slab model for perovskite calculations. In the case of structure relaxation, the convergence criterions were converged to 0.03 eV Å^−1^ and 10^−5^ eV for the residual force and energy, respectively. The Brillouin zones were sampled by a 3 × 3 × 1 Monkhorst–Pack k‐point mesh. To avoid the interaction between two periodic cells, a vacuum space of 15 Å was applied. The formation energy of F vacancy (*E*_Fv_) could be expressed as the Equation (5):

(5) $$E_{\mathrm{Fv}}=E_{\mathrm{deff}}+E_{\mathrm{HF}}-E_{\mathrm{perf}}-1/2E_{H2}$$

where *E*_deff_ and *E*_perf_ are the energies of defective crystal (with one F vacancy) and perfect crystal, *E*_HF_ and *E*_H2_ denote the energies of the HF and H_2_ molecule in the gas phase.

Considering surface reconstruction on perovskite fluorides, the real active sites of NiOOH (0001) surface were thereby modelled for the catalytic reaction. Moreover, the partial replacement of Ni by Fe was conducted to shed light on the effect of Fe substitution. To investigate OER mechanism, the free energies of reaction intermediates, including OH*, O* and OOH*, were systematically calculated based on the computational hydrogen electrode (CHE) model. The free energy change (Δ*G*_ads_) of each elementary reaction could be determined by the Equation (6):

(6) $$\Delta G_{\mathrm{ads}}=\Delta E_{\mathrm{ads}}+\Delta E_{\mathrm{ZPE}}-T\Delta S$$

where Δ*E*_ads_, Δ*E*_ZPE_, Δ*S* and *T* represent the adsorption energy changes of adsorbates, the zero energies, the entropy change and room temperature, respectively.

## Conflict of Interest

The authors declare no conflict of interest.

## Supporting information

Supporting Information

## Data Availability

The data that support the findings of this study are available in the supplementary material of this article.
