# Supplementary material for: Probing Dynamic Self‐Reconstruction on Perovskite Fluorides toward Ultrafast Oxygen Evolution
Source: Adv Sci (Weinh). 2022 Jul 22;9(27):2201916. doi: 10.1002/advs.202201916 (PMC9507342; doi:10.1002/advs.202201916)
Supplement: Supplementary file 1 — Supporting Information [file ADVS-9-2201916-s001.pdf]

ADVANCED SCIENCE
Open Access

Supporting Information

for *Adv. Sci.*, DOI 10.1002/advs.202201916

Probing Dynamic Self-Reconstruction on Perovskite Fluorides toward Ultrafast Oxygen Evolution

Jing Zhang, Yu Ye, Zhenbin Wang, Yin Xu, Liangqi Gui, Beibei He and Ling Zhao**

Supporting Information

Probing Dynamic Self-Reconstruction on Perovskite Fluorides towards Ultrafast Oxygen Evolution

Jing Zhang,[1] *Yu Ye,*[2] *Zhenbin Wang,*[3] *Yin Xu,*[1] *Liangqi Gui,*[1,4] *Beibei He,*[1,5,*] *and Ling Zhao.*[1,5,*]

[1]Faculty of Materials Science and Chemistry, China University of Geosciences, Wuhan, 430074, China.

[2]State Key Laboratory of Geological Processes and Mineral Resources, China University of Geosciences, Wuhan, 430074, China.

[3]Department of Physics, Technical University of Denmark, 2800 Kongens Lyngby, Denmark.

[4]School of Physical and Mathematical Sciences, Nanyang Technological University, 21 Nanyang Link, 637371, Singapore.

[5]Shenzhen Research Institute, China University of Geosciences, Shenzhen, 518057, China.

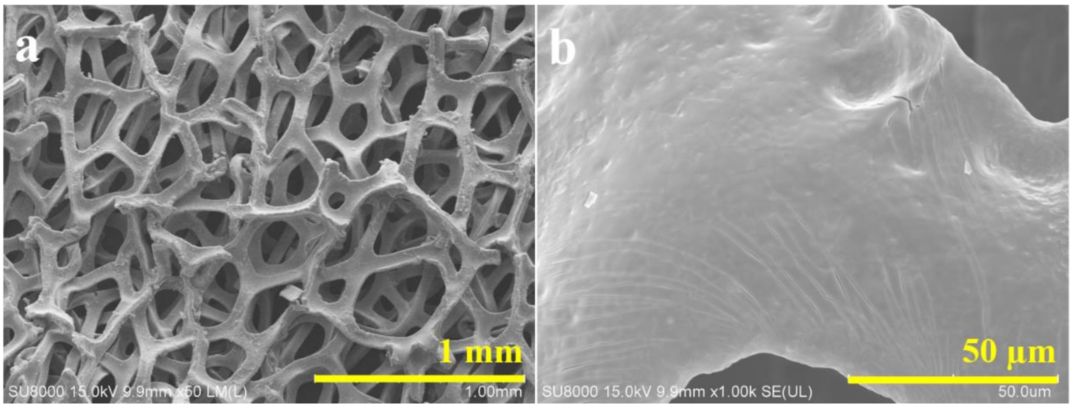

Figure S1. SEM images of the pristine NF.

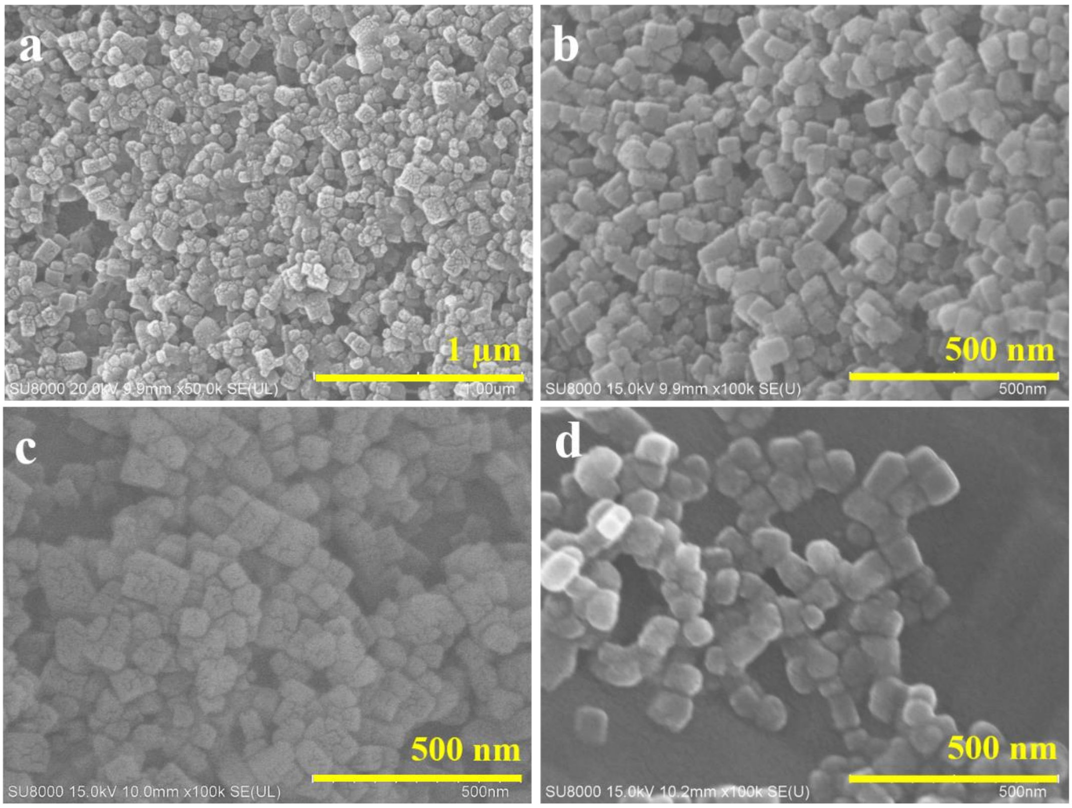

Figure S2. SEM images of (a) KNF@NF, (b) KNFF1@NF, (c) KNFF2@NF, and (d) KNFF3@NF electrocatalysts.

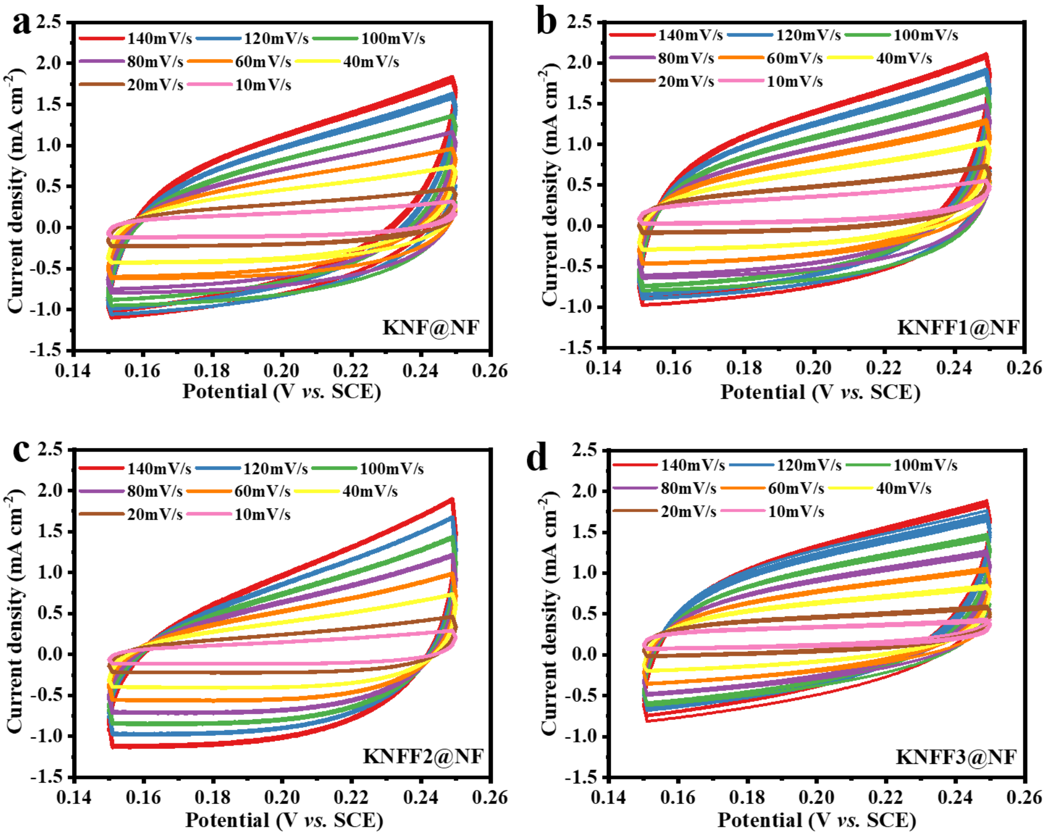

Figure S3. Cyclic voltammograms of (a) KNF@NF, (b) KNFF1@NF, (c) KNFF2@NF, and (d) KNFF3@NF electrocatalysts.

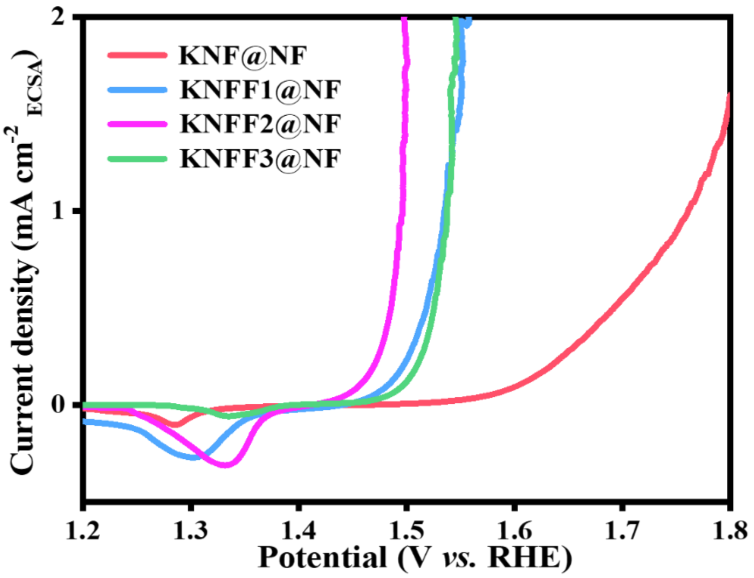

Figure S4. LSV curves with current density normalized to ECSA for KNF@NF, KNFF1@NF, KNFF2@NF, and KNFF3@NF electrocatalysts.

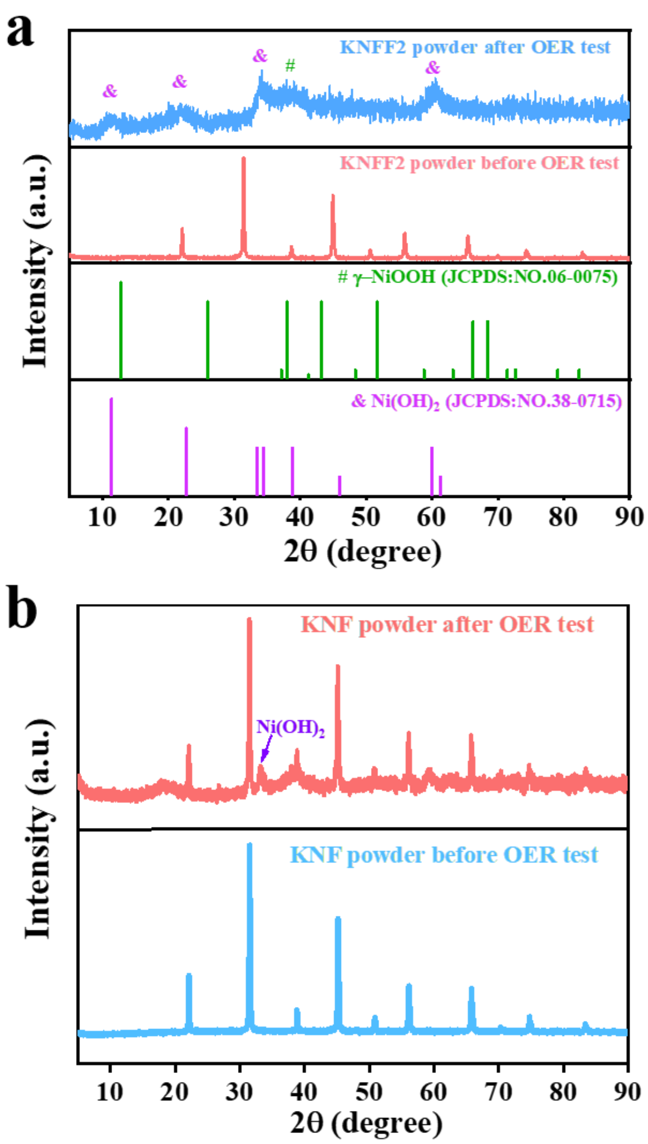

Figure S5. XRD patterns of (a) KNFF2 and (b) KNF electrocatalysts before and after OER operation.

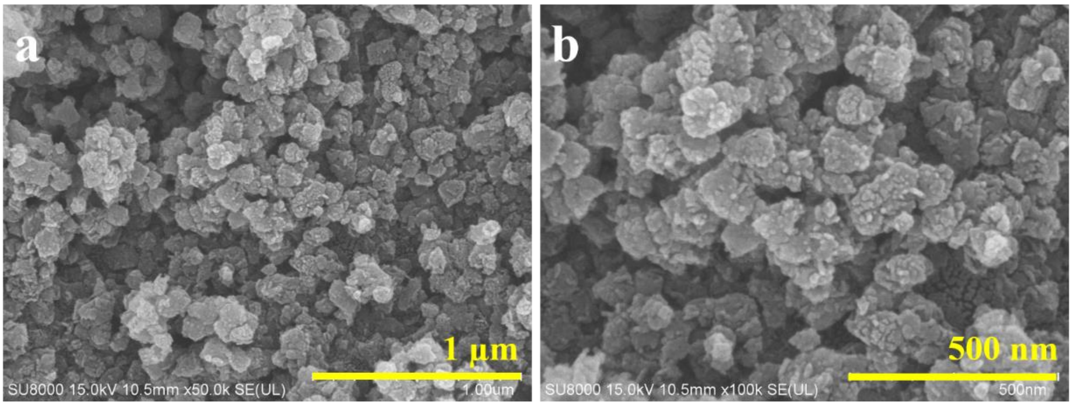

Figure S6. SEM images of KNFF2@NF after OER operation.

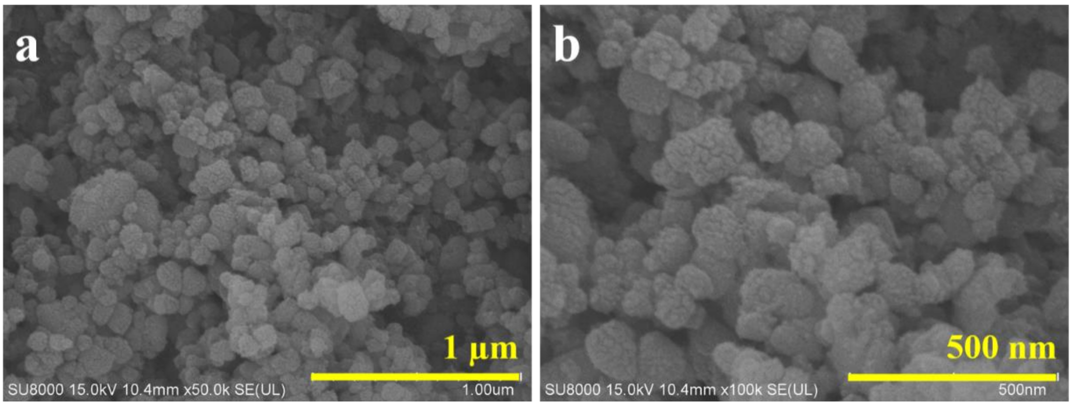

Figure S7. SEM images of KNF@NF after OER operation.

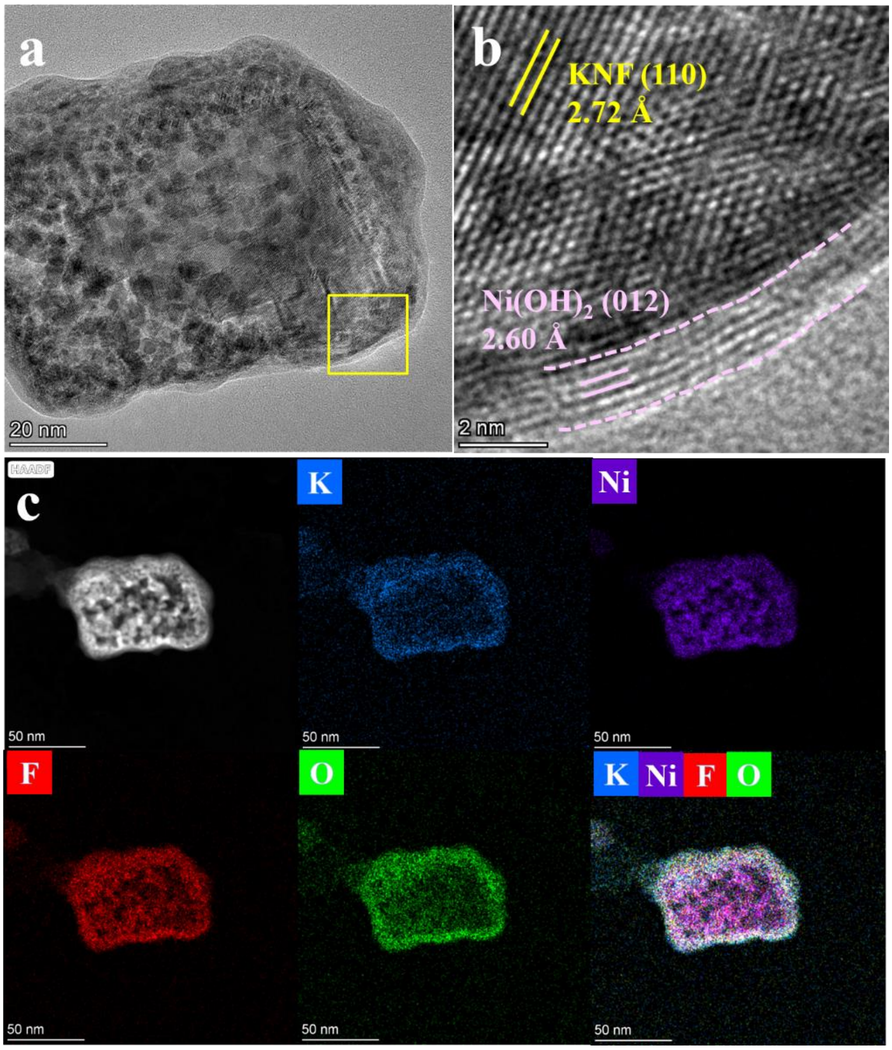

Figure S8. Microstructure of KNF electrocatalyst after OER operation. (a) Low magnification and (b) High magnification HRTEM images; (c) EDS elemental mapping.

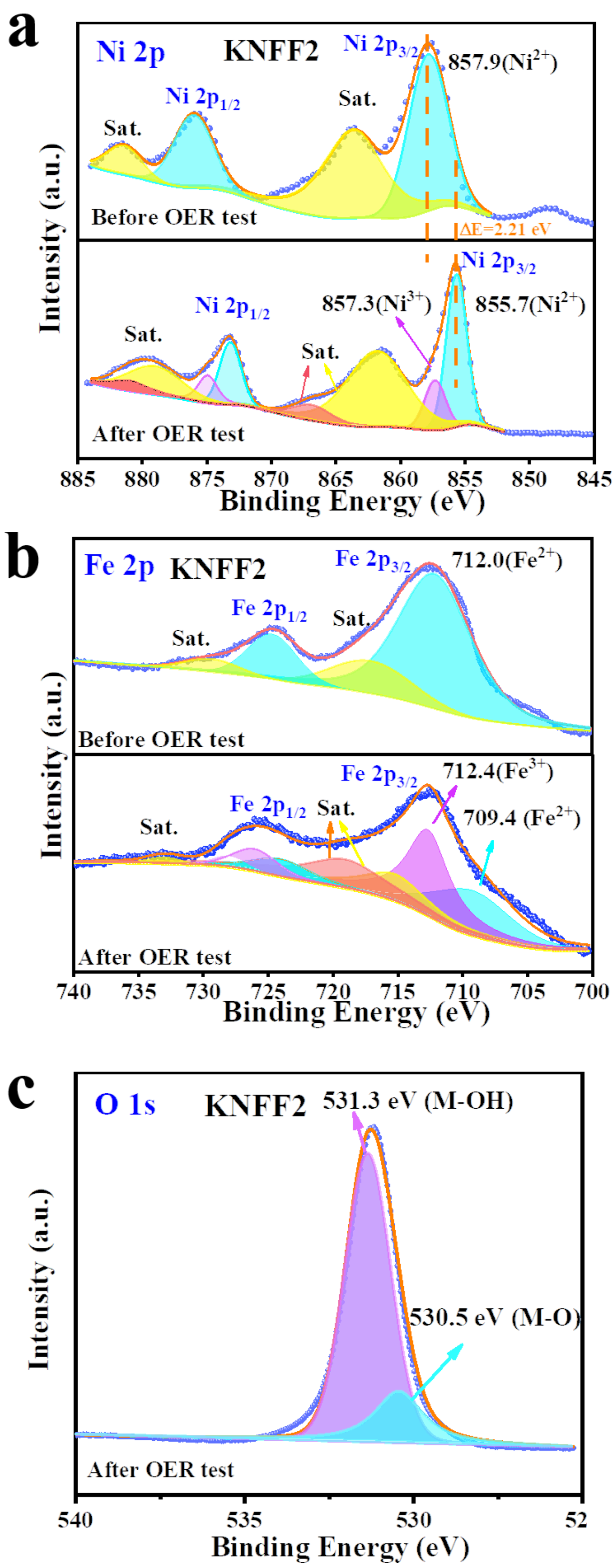

Figure S9. (a) Ni 2p, (b) Fe 2p and (b) O 1s XPS spectra of KNFF2 electrocatalyst

before and after OER operation.

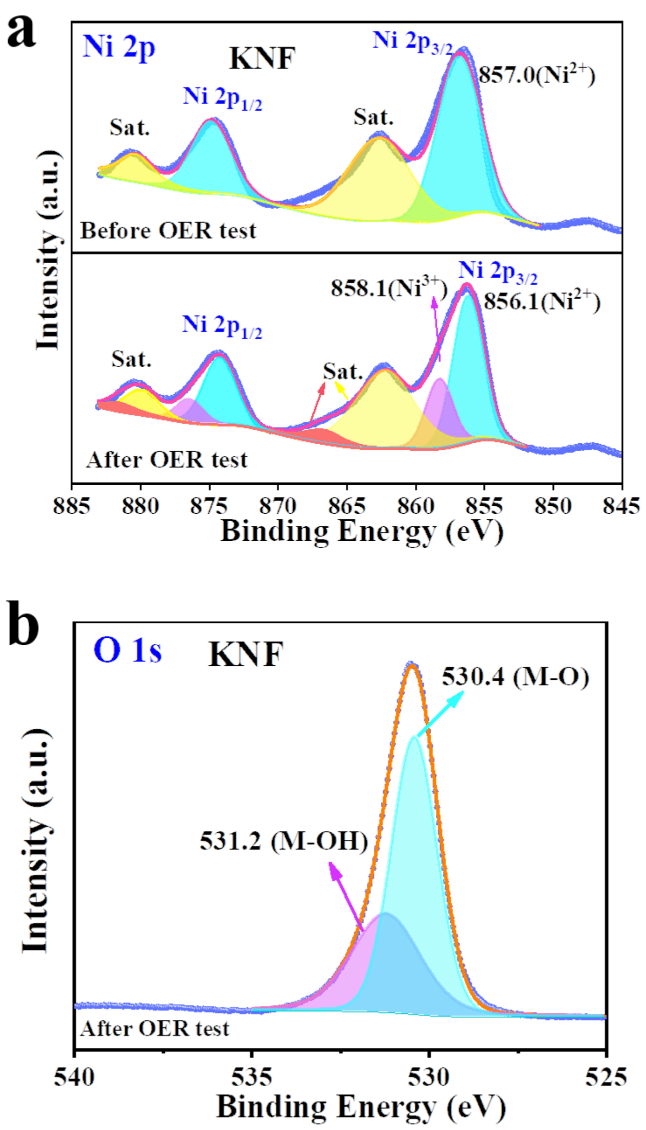

Figure S10. (a) Ni 2p and (b) O 1s XPS spectra of KNF electrocatalyst before and after OER operation.

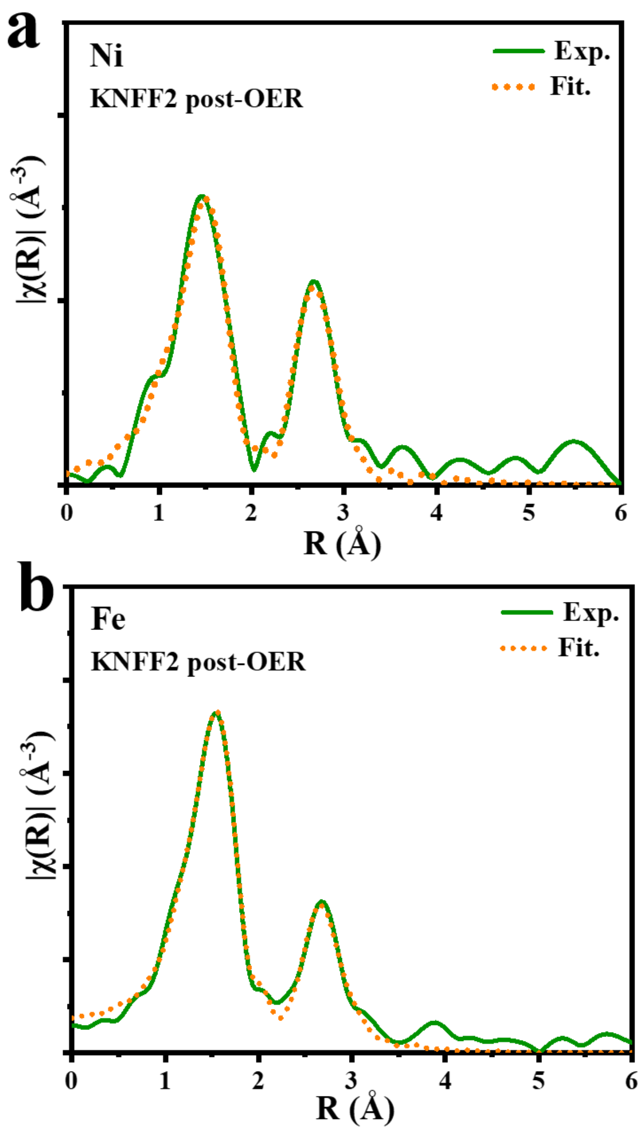

Figure S11. Fitted corresponding structure for KNFF2 after OER in R space: (a) Ni and (b) Fe.

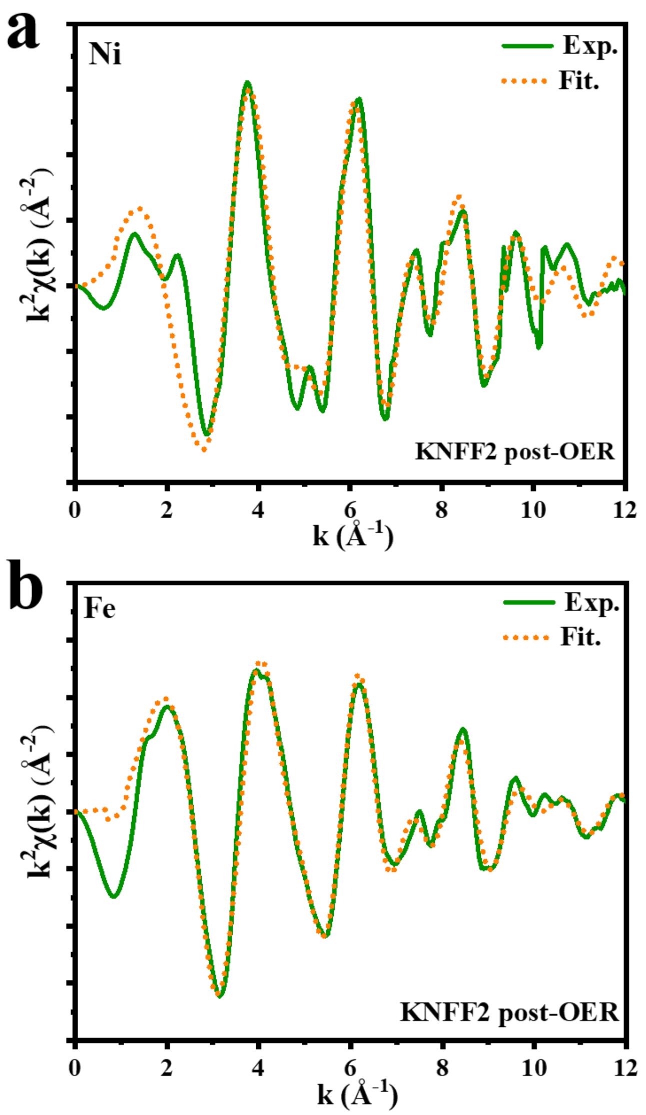

Figure S12. Fitted corresponding structure for KNFF2 after OER in k space: (a) Ni and (b) Fe.

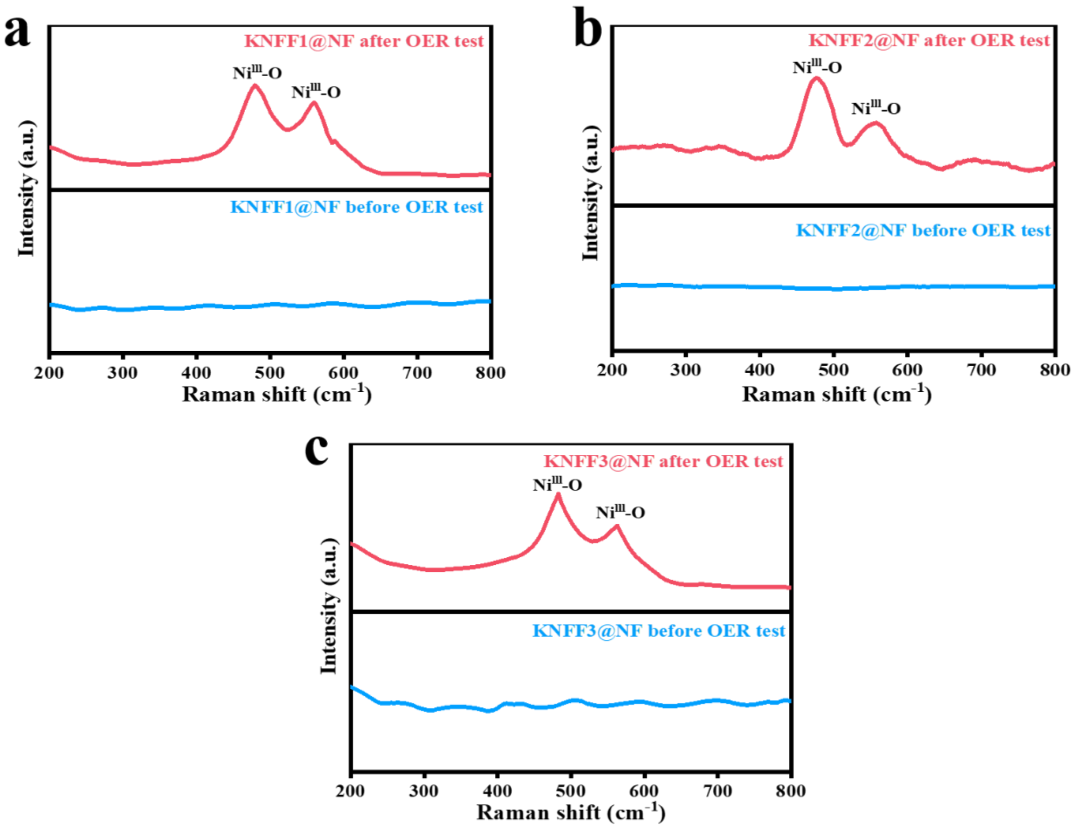

Figure S13. *Ex-situ* Raman spectra of (a) KNFF1, (b) KNFF2, and (c) KNFF3 electrocatalysts before and after OER test.

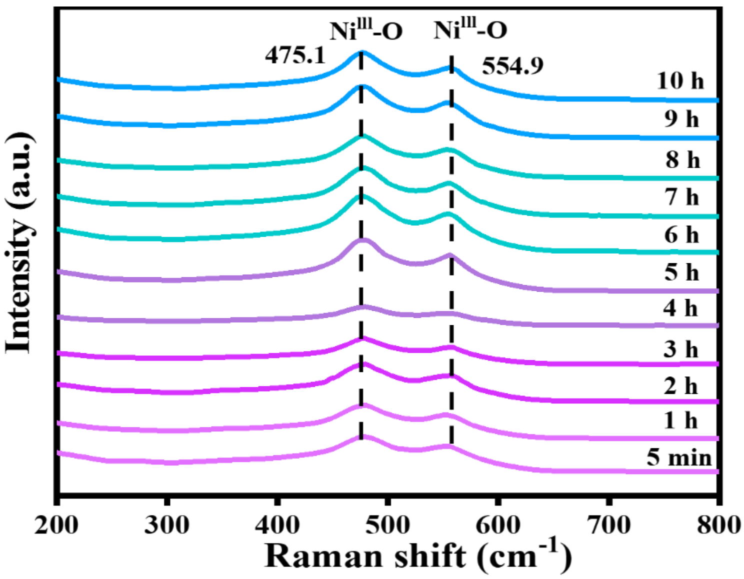

Figure S14. *In-situ* Raman spectra of KNFF2 electrocatalyst during long-term OER operation at 1.53 V *vs.* RHE.

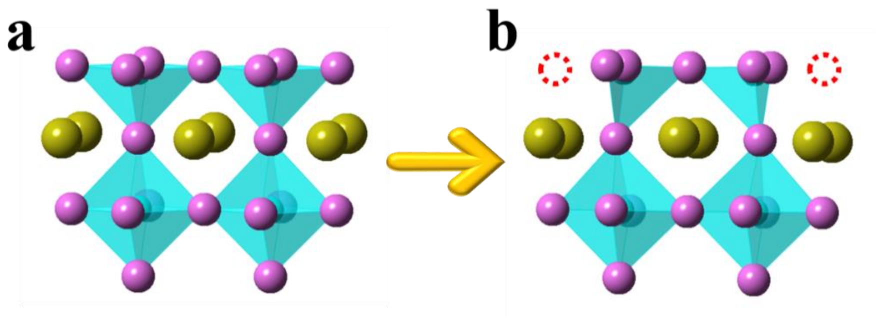

Figure S15. (a) Perfect (100) surface and (b) F defective (100) surface of KNF perovskite (Ni atom: cyan, K atom: yellow, F atom: pink).

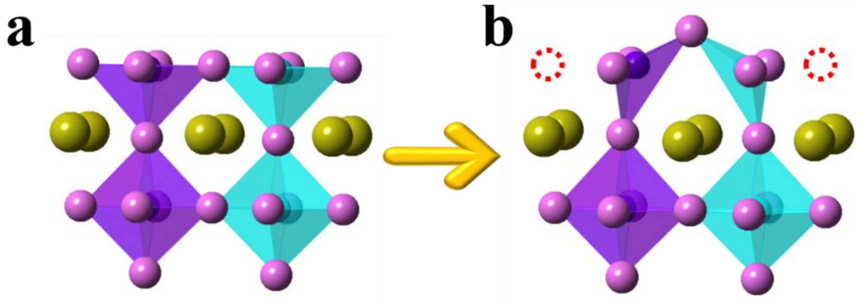

Figure S16. (a) Perfect (100) surface and (b) F defective (100) surface of KNFF2 perovskite (Ni atom: cyan, Fe atom: purple, K atom: yellow, F atom: pink).

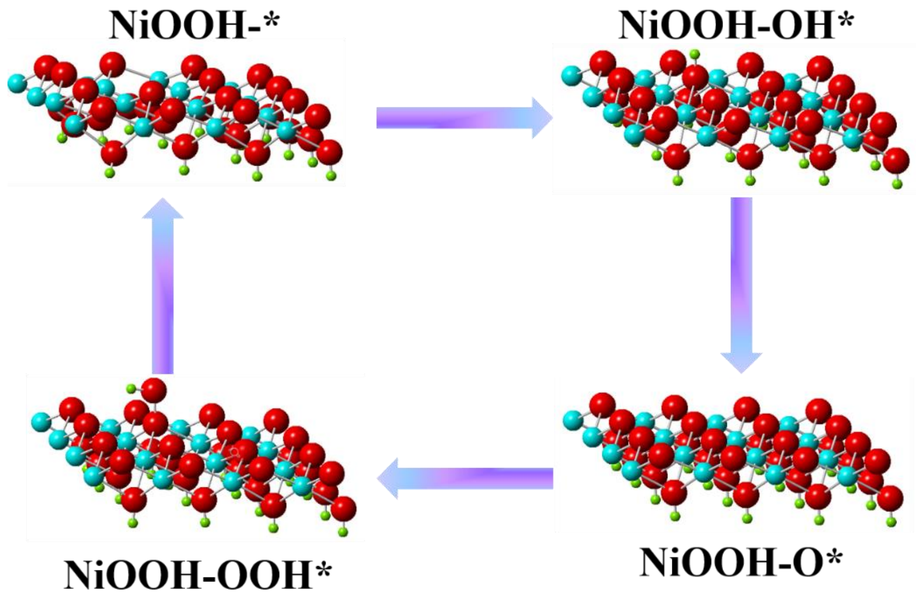

Figure S17. OER pathway: adsorption configuration of oxygen intermediates on γ-NiOOH (Ni atom: cyan, O atom: red, H atom: green).

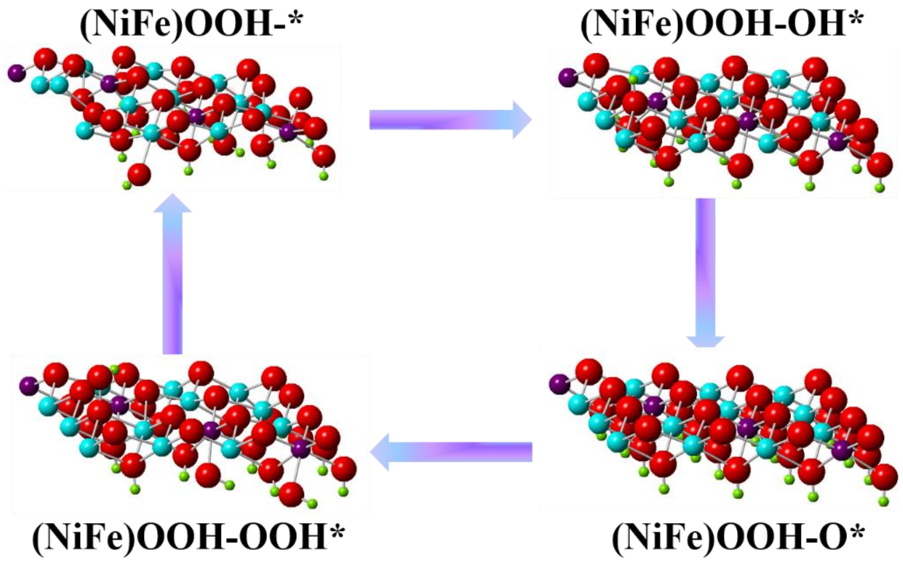

Figure S18. OER pathway: adsorption configuration of oxygen intermediates on γ-(NiFe)OOH. (Ni atom: cyan, Fe atom: purple, O atom: red, H atom: green)

Table S1. Atomic concentration of Ni:Fe in perovskite fluorides determined via ICP-OES.

Electrocatalysts	Ni (Atomic ratio)	Fe (Atomic ratio)
KNFF1	90.93	9.07
KNFF2	80.25	19.75
KNFF3	70.88	29.12
KNFF1 post OER	90.77	9.23
KNFF2 post OER	81.64	18.36
KNFF3 post OER	71.84	28.16

Table S2. Comparison of OER performance with other excellent electrocatalysts.

Catalyst	Overpotential (mV) at *j*=100mA/cm^2	Stability	Ref
**KNFF2@NF**	**258**	**100 h at 100 mA cm^{-2}**	**This work**
γ-FeOOH@NF	335	24 h at 50 mA cm^{-2}	Advance Materials. 2021, 33(11): 2005587 [1]
γ-NiOOH/NiGe@NF	340	21d at 1.48V (vs RHE)	Angewandte Chemie International Edition. 2021, 60(9): 4640-4647 [2]
γ-NiOOH/NiFeGr@NF	322	24 h at 100 mA cm^{-2}	Energy & Environmental Science. 2020, 13(11): 4225-4237 [3]
$NiFe_2O_{4-x}$/NMO-25@NF	304	40 h at 1.65V (vs RHE)	Applied Catalysis B: Environmental, 2021, 286: 119857 [4]
Fe–NiO@NF	415	12 h at 60 mA cm^{-2}	Nano Energy, 2019, 66: 104118. [5]
γ-NiOOH/NiNPS	305	35 h at 1.56V (vs RHE)	Angewandte Chemie International Edition, 2019, 58:17458-17464. [6]
Ni-Fe LDH	327	10 h at 10 mA cm^{-2}	Angewandte Chemie International Edition, 2018, 57(1): 172-176. [7]
FeOx@hcp Ni	314	20 h at 20 mA cm^{-2}	Applied Catalysis B: Environmental, 2021, 284: 119687 [8]
$K_{0.8}Na_{0.2}(MgMnFeCoNi)F_3$	314 at 10 mA cm^{-2}	10 h at 10 mA cm^{-2}	Journal of American Chemical Society, 2020, 142: 4550-4554. [9]
$La_{0.9}Ce_{0.1}NiO_3$	290 at 10 mA cm^{-2}	48 h at 1.6 V (vs RHE)	Advanced Energy Materials, 2021, 11, 2003755. [10]
$LaFe_{0.2}Ni_{0.8}O_3$	304 at 10 mA cm^{-2}	20 h at 1.53V (vs RHE)	Angewandte Chemie International Edition, 2019,58(8): 2316-2320. [11]

Table S3. Fitting parameters of the equivalent circuit for EIS curves, where R_o, R_1, R_2, represent the solution resistance, electrode texture and charge transfer resistances, respectively.

	KNF@NF	KNFF1@NF	KNFF2@NF	KNFF3@NF
R_o/Ω	2.05	2.16	2.21	2.30
R_1/Ω	0.44	0.43	0.19	0.25
R_2/Ω	19.8	2.77	1.39	1.44

Table S4. EXAFS Fitting parameters of KNFF2 and KNFF2 after OER

Catalysts	Path	C.N.	R (Å)	$\sigma^2 \times 10^3$ ($Å^2$)	ΔE	R factor
Ni-KNFF2	Ni-F	6.0±0.5	1.99±0.01	5.1±1	-7.66±1.12	0.016
Fe-KNFF2	Fe-F	5.5±0.7	2.03±0.01	3.3±2	1.40±1.81	0.013
Ni-KNFF2 after OER	Ni-O	7.4±1.0	2.00±0.01	8.1±2	-6.90±1.73	0.016
	Ni-M	5.7±1.6	3.08±0.02	8.4±2	2.90±2.43	
Fe-KNFF2 after OER	Fe-O	6.3±0.4	1.99±0.01	7.6±1	1.74±0.61	0.010
	Fe-M	4.7±1.1	3.08±0.01	9.8±6	4.65±1.76	

S_0^2 fixed at 0.85 and 0.82 for Ni and Fe, respectively.

References:

[1] Wang ke, Du Hongfang, He Song, Liu Lei, Yang Kai, Sun Jinmeng, Liu Yushang, Du Zhuzhu, Xie Linghai, Ai Wei, and Huang Wei. Kinetically Controlled, Scalable Synthesis of γ-FeOOH Nanosheet Arrays on Nickel Foam toward Efficient Oxygen Evolution: The Key Role of In-Situ-Generated γ-NiOOH. Advance Materials. 2021, 33(11): 2005587.

[2] Menezes Prashanth W., Yao Shenglai, Beltrán-Suito Rodrigo, Hausmannn J.Niklas, Menezes Pramod V., and Driess Matthias. Facile Access to Active γ- NiOOH Electrocatalyst for Durable Water Oxidation Derived From an Intermetallic Nickel Germanide Precursor. Angewandte Chemie International Edition. 2021, 60(9): 4640-4647.

[3] Bo Xin, Hocking Rosalie K., Zhou Si, Li Yibing, Chen Xianjue, Zhuang Jincheng, Du Yi, and Zhao Chuan. Capturing the Active Sites of Multimetallic (Oxy)hydroxides for the Oxygen Evolution Reaction. Energy & Environmental Science. 2020, 13(11): 4225-4237.

[4] Choi Juhyung, Kim Daekyu, Zheng Weiran, Yan Bingyi, Li Yong, Lee Lawrence Yoon Suk, and Piao Yuanzhe. Interface Engineered $NiFe_2O_{4-x}/NiMoO_4$ Nanowire Arrays for Electrochemical Oxygen Evolution. Applied Catalysis B: Environmental, 2021, 286: 119857.

[5] Qiu Zhen, Ma Yue, and Edvinsson Tomas. In Operando Raman Investigation of Fe Doping Influence on Catalytic NiO Intermediates for Enhanced Overall Water Splitting. Nano Energy, 2019, 66: 104118.

[6] Huang Jianwen, Li Yaoyao, Zhang Yadong, Rao Gaofeng, Wu Chunyang, Hu Yin, Wang Xianfu, Lu Ruifeng, Li Yanrong, and Xiong Jie. Identification of Key Reversible Intermediates in Self-Reconstructed Nickel-Based Hybrid Electrocatalysts for Oxygen Evolution. Angewandte Chemie International Edition, 2019, 58:17458-17464.

[7] Yu Le, Yang Jingfan, Guan Buyuan, Lu Yan, and Lou Xiaowen. Hierarchical Hollow Nanoprisms Based on Ultrathin Ni-Fe Layered Double Hydroxide Nanosheets with Enhanced Electrocatalytic Activity towards Oxygen Evolution. Angewandte Chemie International Edition, 2018, 57(1): 172-176.

[8] Yan Wenxiu, Shen Yongli, An Chao, Li Lina, Si Rui, and An Changhua. FeO_x Clusters Decorated hcp Ni Nanosheets as Inverse Electrocatalyst to Stimulate Excellent Oxygen Evolution Performance. Applied Catalysis B: Environmental, 2021, 284: 119687.

[9] Wang Tao, Chen Hao, Yang Zhenzhen, Liang Jiyuan, and Dai sheng. High-Entropy Perovskite Fluorides: A New Platform for Oxygen Evolution Reaction. Journal of American Chemical Society, 2020, 142: 4550-4554.

[10] Sun Yu, Li Ran, Chen Xiaoxuan, Wu Jing, Xie Yong, Wang Xin, Ma Kaikai, Wang Li, Zhang Zheng, Liao Qingliang, Kang Zhuo, and Zhang Yue. A-site Management Prompts the Dynamic Reconstructed Active Phase of Perovskite Oxide OER Catalysts. Advanced Energy Materials, 2021, 11, 2003755.

[11] Huang Xuaping, Wang Juan, Pi Yecan, Shao Qi, Tan Yueming, Huang Xiaoqing. Double Perovskite $LaFe_xNi_{1-x}O_3$ Nanorods Enable Efficient Oxygen Evolution Electrocatalysis. Angewandte Chemie International Edition, 2019,58(8): 2316-2320.
